# An Update on Semen Physiology, Technologies, and Selection Techniques for the Advancement of In Vitro Equine Embryo Production: Section II

**DOI:** 10.3390/ani11113319

**Published:** 2021-11-20

**Authors:** Morgan F. Orsolini, Stuart A. Meyers, Pouya Dini

**Affiliations:** 1 Department of Population Health and Reproduction, School of Veterinary Medicine, University of California, Davis, CA 95616, USA; mforsolini@ucdavis.edu; 2 Department of Anatomy, Physiology, and Cell Biology, School of Veterinary Medicine, University of California, Davis, CA 95616, USA; smeyers@ucdavis.edu

**Keywords:** stallion, fertility, sperm, assisted reproductive techniques

## Abstract

**Simple Summary:**

In order to improve fertilization and pregnancy rates within artificial insemination or in vitro fertilization techniques in horses, producers may choose to select the best sperm within an ejaculate. In this paper, we review conventional and novel methods of sperm selection.

**Abstract:**

As the use of assisted reproductive technologies (ART) and in vitro embryo production (IVP) expand in the equine industry, it has become necessary to further our understanding of available semen selection techniques. This segment of our two-section review will focus on the selection of spermatozoa based on quality and sex for equine intracytoplasmic sperm injection (ICSI), as well as current and future developments in sperm sorting technologies. Ultimately, novel methods of semen selection will be assessed based on their efficacy in other species and their relevance and future application towards ARTs in the horse.

## 1. Introduction

The use of assisted reproductive techniques (ARTs) is expanding the field of equine reproduction, providing valuable opportunities to produce foals from animals which are unable to breed, conceive, or carry a pregnancy to term, due to either sub-fertility or logistical management issues. Techniques such as artificial insemination (AI) and embryo transfer (ET) are practiced with consistent success, and have already become an indispensable part of equine reproduction [1]. Newer techniques, such as transvaginal oocyte aspiration (TVA) and in vitro embryo production (IVP), are also being implemented worldwide, allowing veterinarians and farm managers to maximize the reproductive performance of horses [1,2,3,4]. Combining IVP with the use of sexed semen, the use of frozen-thawed sperm, observation of early embryo development, utilization of pre-implantation genetic testing, and cryopreservation of produced embryos further expands the potential of IVP in horses, despite some of these methods being in their infancy [5,6,7,8,9,10].

Most of the efforts for optimizing IVP outcomes are focused on oocyte maturation protocols, such as monitoring oocytes for meiotic progression [11,12], microtubule and spindle assembly [13,14], chromosomal abnormalities [15,16,17], nuclear maturation [18,19], and cumulus cell expansion [20,21]. Oocyte competence is critical in cases of subfertility, in part because the number of available oocytes at any given time is significantly less than the number of available sperm cells [11,22]. However, sperm quality should not be overlooked as an indicator of fertilization and development potential, as there are wide ranges of sperm quality within an ejaculate that may influence ideal embryo production [23].

In horses, IVP is currently limited to a technique called intracytoplasmic sperm injection (ICSI) due to the failure of conventional in vitro fertilization (IVF) to produce foals [24]. One of the main advantages of ICSI is the low number of spermatozoa required per procedure; ultimately only a single male gamete needs to be selected for each oocyte. Therefore, ICSI has become the primary technique for producing embryos from men with low sperm count or viability [25]. Likewise, semen availability is one of the main reasons to perform ICSI in horses, allowing for the production of greater numbers of offspring from a reduced number of spermatozoa [26]. In general, frozen-thawed semen from stallions usually contains a limited number of viable spermatozoa and an overall worsened post-thaw quality [27]. However, frozen semen is ideal for ICSI because it can be stored and shipped easily, has a decreased dependency on proximity and frequent collection of stallions, requires only a fraction of a frozen ejaculate to be thawed to obtain sufficient numbers of spermatozoa for fertilization, and allows for the use of a variety of stallion genetics, regardless of whether or not the stallion is healthy or deceased [28]. Additionally, it has been demonstrated that using frozen-thawed sperm for ICSI results in similar fertilization and embryo development rates to fresh semen, with some individual variation [6]. Other studies have shown that freeze-dried or air-dried sperm adequately maintain chromosome integrity and are also capable of producing live offspring after ICSI [29,30,31,32,33,34]. However, little is known about the potential effects of drying on overall sperm competence and embryo development and it is not a standard practice. Therefore, frozen-thawed sperm is the current ideal choice for equine ICSI.

Despite the numerous benefits of ICSI, is important to note that the manual selection of a sperm for ICSI bypasses the natural selection of viable spermatozoa that would naturally occur in the female reproductive tract, and to a lesser extent during conventional in vitro fertilization (IVF) procedures [35]. Consequently, the absence of natural sperm selection may represent a barrier to optimal fertilization and development [35,36]. In particular with ICSI, it is possible to select a visually normal spermatozoa possessing damaged DNA or internal structure, which could lead to abnormal embryo development or miscarriage as observed in humans [37,38]. Therefore, the use of artificial selection techniques to select for the most competent spermatozoa in a sample is a critical step in the optimization of ICSI outcomes.

Sperm selection has become an integral part of ARTs for both humans and animals [39,40]. Spermatozoa from domestic mammals, as well as humans are generally evaluated and selected based on factors associated with their fertilization potential, which is then used to define sperm quality [41,42,43]. Various techniques to select the fraction of “high quality” viable spermatozoa in a sample rely on motility, morphology, DNA integrity, surface charge, and biochemical markers as indicators of potential fertility [44] and will be covered later in this review. However, there is increasing evidence that common selection parameters are insufficient indicators of fertility [41,45]. Conventional sperm selection techniques have been shown to be effective for the positive selection of motile and morphologically normal spermatozoa from a sample, yet they do not directly select for other important factors associated with fertilization and development, such as DNA fragmentation, membrane integrity, and spermatozoal ultrastructure [39,46,47,48]. Although conventional selection methods for the enrichment of motile, morphologically normal spermatozoa within a population has been correlated with improved DNA integrity and consequently improved fertilization, blastocyst, and pregnancy rates, there is still room for improving the margin of error within sperm selection techniques [49,50].

This section (Section II) of our review will focus on the ultimate selection of spermatozoa through advanced ARTs. Methods of sperm selection for ICSI based on both quality parameters and sex-chromosome will be presented and assessed based on their efficacy in horses. Ultimately, novel sperm selection methods used in non-equine species will be discussed in regard to their future application towards ARTs in the horse.

## 2. Sorting Semen: Significance and Method

In vivo, it is believed that sperm are naturally “selected” as they navigate through the female reproductive tract, resulting in only the most competent spermatozoa reaching the oviduct and ultimately fertilizing an ovum [35,36]. However, these natural sorting procedures are bypassed during IVP, which could contribute to the suboptimal outcome of these techniques. According to a meta-analysis carried out in humans, IVP is only about one-fifth as efficient as natural reproduction due to damage from cryopreservation and overall worsened gamete competence [51]. Inefficiencies of IVP have been documented in many species, beginning with the ability of in vitro culture conditions to disturb fertility and developmental competence [24,52,53,54,55]. In addition, many have reported specific issues with fertilization and development events. For example, in cattle, fertilization rates after ICSI are extremely low potentially due to a failure in pronuclear formation without supplemental activation or due to physical disruption from the ICSI procedure [56,57,58,59]. In porcine IVF there is a high rate of polyspermia due to a reduced ability of in vitro-matured oocytes to invoke their mechanism of zona blocking against polyspermia [60,61]. In horses, only two cases of IVF have been reported; this lack of success in conventional IVF is most commonly attributed to the inability of the sperm to penetrate the thick zona of the oocyte due to incomplete capacitation [24,62]. Thus, ICSI is the only practical method of IVP in horses [5]. Despite the concentrated efforts in refining equine ICSI, blastocyst and pregnancy rates leave room for improvement [1]. Within a single study, reported blastocyst rates varied from 10–70%, dependent on the source of oocytes (pre-ovulatory oocyte vs. immature), oocyte maturation protocols, and culture conditions [1,63]. Therefore, blastocyst, pregnancy, and foaling rates are subject to significant improvement and standardization across the equine industry.

Although it is possible to produce healthy embryos and offspring from low quality sperm samples, it is preferable to process and select morphologically and functionally superior sperm to maximize the chances of successful fertilization and embryo development [40,64]. It is also noteworthy that there is limited information regarding the relationship between “good quality” sperm parameters and fertility [40]. Conventional sperm selection techniques generally rely on assessment of motility and morphology, which are factors that are positively correlated with fertilization and pregnancy rates for IVP [65,66,67,68]. However, spermatozoa with good motility and morphology will not always exhibit optimal viability, and may instead have poor DNA integrity, apoptotic factors, and disturbed mitochondrial integrity [48,69]. Additionally, centrifugation steps required for specific processing methods are capable of generating injurious levels of reactive oxygen species (ROS) and ultrastructural damage in mammalian sperm [39,69,70,71]. Thus, advancements in selection methods are likely to reduce sperm injury and to improve fertilization and pregnancy outcomes.

**Figure 1:**
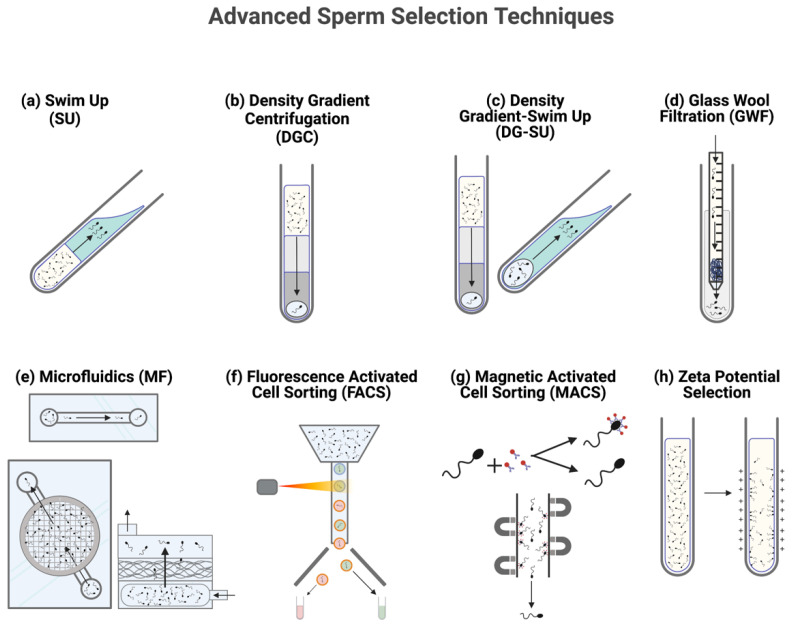
Conventional and novel methods of sperm selection. Selection techniques used to select superior quality sperm include (**a**) Swim Up (SU), (**b**) Density Gradient Centrifugation (DGC), (**c**) Density Gradient-Swim Up (DG-SU), (**d**) Glass Wool Filtration (GWF), (**e**) Microfluidics sorting (MF), (**f**) Fluorescence Activated Cell Sorting (FACS), (**g**) Magnetic Activated Cell Sorting (MACS), and (**h**) Zeta Potential Selection.

Many sperm selection methods have been developed in order to maximize the chances of selecting highly viable spermatozoa with variable success (Figure 1, Table 1) [40]. Commonly accepted and practiced methods of sperm selection include swim up (SU) and density gradient centrifugation (DGC) [39,40,47]. SU and DGC are widely used due to their simplicity and cost efficiency, and are known to select for improved motility, morphology, and nuclear maturity in a semen sample [39,47].

### 2.1. Density Gradient Centrifugation

DGC works by overlaying a single or double density colloid gradient (known as a continuous or discontinuous gradient, respectively) with a semen sample of mixed quality within a centrifuge tube [39,72,73]. The entire tube is then spun at a moderate *g*-force (300–600× *g*) for 15–30 min in order to induce passage through the gradient and separation of the high and low quality spermatozoa [39]. In a double density gradient, the less dense, upper layer will filter out larger macromolecules, leukocytes, or other unwanted cellular debris [74]. Mature spermatozoa should be able to easily pass through the upper gradient, and upon reaching the second, denser colloid layer, the morphologically normal spermatozoa will possess a greater density and will be able to orient head-downwards, allowing them to swim downwards through the colloidal silicon gradient and form a pellet with the aid of centrifugation forces [39,74,75]. Percoll^®^ DGC is the most common gradient in most animal industries but is prohibited in human sperm preparation due to potential inflammatory, ultrastructural, and endotoxic effects of the PVP-coated silica particle, which is associated with cytoplasmic fragmentation and worsened embryo development [76,77,78,79,80]. In equine reproduction, the use of Equipure^™^ has become a common substitute for Percoll^®^ in various laboratories. Results from Equipure^™^ centrifugations have yielded enriched motility, morphology, and pregnancy rates as compared to other centrifugation methods [81,82,83]. It has been shown that Equipure^™^ not only selects for progressively motile spermatozoa, but also enriches the population of viable sperm with good mitochondrial membrane integrity from frozen-thawed samples [84]. The resulting pellet after either Percoll^®^ or Equipure^™^ centrifugation has been generally known to enrich the population of motile, morphologically normal spermatozoa with an intact genome in both men and stallions [81,83,85,86]. Alternatively, some report DNA damage to actually increase as a result of centrifugation in horse or human [75,86]. Results likely vary due to individual, handling, or protocol variations. Increased DNA damage can lead to poor embryo quality, blastocyst rates, implantation rates, and pregnancy to term rates after IVP, and thus it is critical to understand the potential for inducing damage with centrifugation protocols [87,88].

### 2.2. Swim Up

In contrast to DGC, the SU procedure does not require a centrifugation step, and relies on the inherent progressive motility of sperm to swim upwards through a medium over a period of 30 to 60 min, with the top fraction ultimately being selected [39,48]. SU has been shown to select highly motile, morphologically normal, DNA-intact populations from human sperm samples, while also removing extraneous cell or protein debris [39,40,48,89,90]. Unfortunately, the recovery rates of SU are low, meaning SU is only a viable assay for highly concentrated samples or for procedures such as ICSI that do not require a significant number of spermatozoa in the final selected fraction [39,91].

### 2.3. Combination Density Gradient-Swim Up

DGC and SU have also been used in combination (DGC-SU) by pelleting the sperm using DGC and then allowing sperm to swim upwards through an overlying media. DGC-SU has been shown to enrich for motility and morphology better than SU alone, to decrease the percentage of ultrastructural abnormalities in the selected sample, as well as select for the population with overall better DNA integrity as compared to DGC alone [92,93]. This method has also been shown to be an effective way of removing pathogens, such as equine arteritis virus or bovine viral diarrhea virus, from contaminated semen while also selecting for undamaged spermatozoa [94,95]. Therefore, this method could have significant benefits for other equine diseases transmissible through an ejaculate.

### 2.4. Glass Wool Filtration

Glass Wool Filtration (GWF) is another technique that has been used in both human and animal reproduction [71]. This method is intended to mimic the ability of the female reproductive tract to effectively filter out dead sperm, leukocytes, and infectious materials, by allowing spermatozoa to swim through and be filtered by a dense arrangement of glass wool fibers within a column [71,96]. In humans, spermatozoa separated with GWF yield a sample enriched for motility, morphologically normal spermatozoa, and good chromatin condensation, as well as having a high recovery rate and good cleavage and blastocyst rates [97,98]. GWF was also shown to produce a sufficient number of cells, with better recovery than SU, for insemination with frozen-thawed spermatozoa [99]. In an experiment with bovine sperm comparing DGC and GWF, GWF was capable of enriching motility, membrane integrity, and GWF-selected sperm used for IVF resulted in higher cleavage and blastocyst rates than control samples [100]. In horses, the pregnancy rate from deep-horn inseminations with sperm separated by GWF was elevated above the rate when using an absolute number of sperm, and similar to that of insemination with Percoll^®^ separated sperm, showing that GWF is a valuable technique for many species [101]. It is noteworthy that some reports claim that traditional GWF can damage the sperm head and acrosome ultrastructure [102], and glass wool fibers could appear in the filtered product, which poses a problem for artificial insemination procedures [103].

All of the techniques described thus far work on the basis of selecting highly motile, morphologically normal, and intact spermatozoa, and are moderately successful in doing so. However, the ability of these techniques to improve motility and morphology parameters does not always correlate to the selection of spermatozoa with the best DNA integrity and overall fertilization potential [48]. In response, other techniques have been developed in order to select sperm based on viability and biophysical markers.

### 2.5. Fluorescent Activated Cell Sorting

Flow cytometry has been utilized in order to characterize various quality parameters within an ejaculate, including membrane integrity, ROS generation, capacitation, acrosome reaction, mitochondrial status, apoptotic markers, and DNA integrity [104]. Although general characterization of a cell population by flow cytometry requires the use of a fixative, fluorescence activated cell sorting (FACS), has been adapted in order to recover live cells. FACS utilizes a variety of fluorescence stains and dyes which are biologically compatible or conjugated to bind to sperm based on specific sorting parameters and primarily works by allowing for the removal of damaged cells from a sample (negative selection). The cell’s affinity for the stain or dye allows for it to emit a readable fluorescent or non-fluorescent signal (more complex probes can be sorted based on the degree of fluorescence) when the cell is excited by a laser and the probe is activated. Subsequently, the droplet is charged and sorted into an appropriate subpopulation by deflection plates [71]. The use of a stain or dye targeting indicators of poor sperm quality results in the negative selection of the high-quality population which can then be used for a variety of in vitro procedures. Live sperm have been flow-sorted based on membrane permeability and apoptotic markers [105,106,107], mitochondrial membrane potential [108], and even sex chromosomes [109].

One of the early stages of apoptosis involves a change in the membrane where phospholipid phosphatidylserine is transferred to the sperm’s outer membrane and displaces phosphatidylcholine [110,111]. A method to enrich the population of non-apoptotic spermatozoa in a sample is to tag and remove apoptotic spermatozoa from the population by FACS with fluorescently labeled Annexin-V; the antigen of phosphatidylserine [105,106]. Sperm negatively selected via Annexin-V FACS have been shown to possess intact chromatin [107]. Additionally, in a human clinical trial, spermatozoa from the population that did not bind Annexin-V during FACS were used for ICSI resulted in improved pregnancy and live birth rates, as well as reduced miscarriage rates over embryos generated with sperm selected from SU [112].

Sex selection using flow cytometry is perhaps the most common application of FACS to sperm selection and has been successful in separating sperm from many species including humans, cattle, horses, pigs, sheep, goats, dogs, cats, deer, elk, and water buffalo, as reviewed by [113]. In horses, sexed semen has been used to produce live foals, sexed with over 90% accuracy [114,115,116,117]. More information on sex selection can be found below (Sexing Semen).

Despite the advantages of FACS sorting, it has been shown that FACS in equine sperm induces significant oxidative and DNA damage to spermatozoa [118]. In addition, FACS can cause significant mechanical stress due to the high-pressure throughput, and laser and dye exposure may reduce mitochondrial activity and motility, as demonstrated with bovine sperm [119,120]. It can thus be extrapolated that mechanical and functional stress may be induced regardless of specific dyes, laser wavelengths, or flow pressures. Additionally, flow cytometers are costly to maintain and operate, and selection can be time consuming due to the individual characterization of each cell; therefore, flow cytometry is not always an ideal method of selection [115,116,121].

### 2.6. Microfluidic Sorting

Another technique for high quality sperm selection is the microfluidic (MF) sorting method, which can select highly motile sperm based upon rheo-, chemo-, and thermotactic behaviors of viable spermatozoa, while also removing extraneous cellular debris, in an attempt to mimic aspects of in vivo sperm selection [122,123,124,125]. Various MF devices exist, including those that rely on the ability of the highly motile subpopulation to swim through a porous membrane [126,127,128] or combinations of channels and collection chambers to be selected [125,129,130,131,132,133,134]. MF sorting with human and bovine sperm has shown to select for sperm with overall enriched motility, viability, and DNA integrity, as well as reduced ROS generation, when compared to centrifugation methods [126,127,132].

In porcine IVF, polyspermia is a large contributor to developmental incompetence in early embryos; thus, a microfluidic-IVF combination device known as a Microfluidic Sperm Sorter (MFSS) has been developed to combine the sperm selection and IVF processes [130]. In a study by Sano et al., (2010), MFSS-generated embryos showed reduced cases of polyspermia and improved developmental competence as compared to embryos generated by traditional IVF [130]. This method has also been used in cattle IVF to improve developmental competence and blastocyst rates [131]. Other variations among microfluidic devices include the use of hydrostatic pressures to facilitate rheotactic behaviors [133] or highly viscous medias [134] to more closely mimic in vivo fertilization.

In a recent study with horse sperm, microfluidic devices containing a porous membrane that allows only motile spermatozoa to swim through resulted in a selected population enriched for sperm with normal morphology, and improved motility, viability, and DNA integrity parameters [128]. This study also showed that MF yielded similar results to DGC and was superior to results from SU, but no clinical outcomes were investigated [128]. Yet, despite the widely reported benefits of MF sorting, it has also been demonstrated that some microfluidics may impose stress upon boar, but not bull, spermatozoa and negatively impact viability [135]. As boar spermatozoa are often used as a model for human spermatozoa, this phenomenon, as well as potential injuries to spermatozoa from other species, requires further investigation.

### 2.7. Magnetic Activated Cell Sorting

Another relatively new technique for selection of viable spermatozoa is the use of magnetic nanoparticles (MNP) to select for various parameters of quality, otherwise known as Magnetic Activated Cell Sorting (MACS) [136,137]. Nanoparticles, defined as being less than 100 nm in diameter, can be coated with a variety of magnetic compounds and subsequently conjugated to a variety of biomarkers for physicochemical properties of the sperm [138,139]. Applications of the iron oxide (Fe_3_O_4_) MNP are diverse, and consequently this is a common choice of magnetic conjugate [138,140]. Magnetized biologically relevant conjugates can be incubated with spermatozoa and then passed through a magnetic field for sorting [136,137]. Previously, MACS has been used for high quality sperm selection with samples from humans, pigs, cows, and donkeys by selecting for characteristics of apoptotic and prematurely acrosome reacted sperm, which results in improved fertilization and embryo development [136,137,139,141,142,143].

Specifically, Annexin-V conjugated MNP have been used to eliminate human spermatozoa in the early stages of apoptosis from a population using a paramagnetic microbead conjugated to Annexin-V in order to bind to phosphatidylserine and negatively select for an intact population [137,143]. In early studies, several groups were able to successfully reduce the percentage of apoptotic sperm within their sample without any observable negative effects [137,144]. Later studies further found that Annexin-V MACS prior to cryopreservation resulted in significant improvements in survival, motility, and mitochondrial integrity after thawing as compared to an untreated control [142,143]. Paasch et al. (2003) compared binding between known infertile patients and donors and found that infertile patients had much higher binding rates to the Annexin-V MNP with strong specificity for apoptotic cells. In another human clinical trial, Annexin-V MACS and DGC selection combined was the most optimal method of selecting sperm with improved motility, viability, and morphology, and a reduction in early apoptotic markers, over that of DGC alone or MACS alone [145].

In animals, MACS has been used in pigs to remove both apoptotic and acrosome reacted spermatozoa via MNP conjugated with Annexin-V and Lectin, respectively [139]. MACS selection resulted in an enriched motile population, and no negative effects have been observed when negatively selected sperm were used for AI, which negates concerns over potential toxicities of nanoparticles in sows [139,146]. In donkeys, peanut agglutinin (PNA)-lectin conjugated nanoparticles have been used to remove acrosome damaged spermatozoa, while simultaneously improving progressive motility, and in some cases membrane viability [141]. Additionally, MACS has been proposed as an alternative to flow cytometry sex sorting and has been successfully used to enrich the population of X spermatozoa with 90% accuracy [147]. MACS sex-sorted semen also demonstrated good viability and motility without premature capacitation or DNA damage [147]. MACS has not been widely used in the equine breeding industry but may be suitable for stallions with subfertility.

### 2.8. Zeta Potential Selection

Another novel sperm selection technique is the zeta potential sorting method. In humans, a greater net negative zeta potential, has also been reported in mature, morphologically normal, DNA intact sperm, thus making zeta potential a potential marker of sperm quality, or fertilization potential as well as playing a functional role in in vivo selection [148,149,150,151,152,153,154,155,156]. As described in Section I of this review, zeta potential is the electrostatic potential at the slipping plane of the cell and is an estimation of the surface charge of the cell [152,157]. Due to the nature of zeta potential measurements being dependent on fluid dynamics, there is no defined optimal measurement for this parameter. Therefore, zeta potential measures must be performed under identical conditions in order to be compared. For example, a sample with a greater net negative charge is theorized to be better quality than a sample with a more positive zeta potential under identical conditions. Thus, membrane charge is both a revealing and complex trait to accurately measure and interpret.

Regardless of complexity, several selection methods have been developed in order to separate sperm based on membrane surface charge [151,154,156]. An existing zeta potential-based selection method involves inducing a positive charge on a glass centrifuge tube using friction and allowing the more negatively charged spermatozoa to bind. This method has been used in human IVF to successfully select sperm with overall improved DNA integrity, morphology, and protamine content compared to unprocessed semen [153,155]. In turn, this resulted in improved fertilization and pregnancy rates [150,153,154,156]. In another study, selection of human spermatozoa based on morphology, motility, and viability resulted in a significantly increased net negative charge of the sample; for example, morphologically normal semen possessed an average zeta potential of −7.79 mV whereas morphologically abnormal semen read an average −5.37 mV [149]. An alternative method of zeta quality selection utilizes electrophoresis to drive the movement and isolation of high-quality spermatozoa that also possess a greater net negative charge. Subsequently, this method is able to improve measures of morphology, and select against spermatozoa with DNA damage, although motility and viability parameters did not change significantly from the original sample [150]. The selection of high-quality equine semen based on zeta potential has yet to be reported, and could have pronounced effects on outcomes of equine IVP.

## 3. Sexing Semen

Selection of spermatozoa based on the presence of an X or Y chromosome is of significant interest for many species [116]. Although not used in human applications, sex selection is especially important in equine and agricultural industries where female or male phenotypes may be better suited for sporting or production outcomes, as well as appealing to owner preferences. In horses specifically, female or male phenotypes are desirable in different forms of recreation [116]. For example, females are used almost exclusively in Polo sports and are also the preferred sex to be used as cutting horses and in Quarter horse racing [117]. However, males are more desirable as reining horses, and are preferred for Thoroughbred racing, dressage, and show jumping, as well as fetching higher prices at Thoroughbred auctions [116,158,159,160]. In addition, the ability to determine fetal sex may aid in making key breeding management decisions, as well as make it easier to sell pregnancies with known fetal sex for a higher price [116]. Due to the strong preferences of many industries for one sex or the other, it is of key interest to be able to pre-determine sex, primarily starting with sexed semen. However, in order to select for one sex or the other, clinicians must rely on the physiological differences between X and Y chromosome bearing spermatozoa.

Although X and Y spermatozoa are essentially equivalent in regard to functionality, there are notable differences. Most notably, the human X spermatozoa contains approximately 2.8% more genetic material than the Y spermatozoa, and differences for livestock species range 3–4.2% [161]. This principle has become the foundation for sex sorting with flow cytometry.

Currently, flow cytometry is the only vetted method for separation of X and Y chromosome-bearing spermatozoa. Sex sorting with flow cytometry utilizes the Hoescht 33342 fluorescence stain (which preferentially binds to adenine-thymine (AT) rich regions along the minor groove of DNA) to categorize individual sperm based on differences in sex chromosome mass (as reviewed by [113,117,121]). Sperm are individually run through the flow cytometer, and their respective droplet is charged according to relative fluorescence and separated [115]. Sex selection using flow cytometry has been successful in separating X- and Y-bearing sperm from a variety of species (as reviewed by [113]. In horses, sexing technologies have been used to produce live foals, sexed with over 90% accuracy [114,115,116,117].

Although various technical advancements have been made with flow sorting, the method is inefficient in producing doses adequate for AI and can cause damage to the sperm including reduced motility, generation of reactive oxygen species, acrosomal and membrane damage, and reduced longevity of the sperm (as reviewed by [115,117,119,121,162]). This results in pregnancy rates as low as 10–50% [114,158,163,164]. Injury to the sperm cells may be due to a number of the following: staining, high pressure flow, charging, deflection, and specific handling methods. These effects are worsened in frozen-thawed samples, making flow sorting undesirable for procedures such as ICSI, where frozen-thawed samples are primarily used [113,115,116,119,165,166]. Additionally, flow cytometry is expensive and time consuming, making it unappealing or inaccessible to many commercial operations [115,116,121]. In stallions the unique head shape of spermatozoa, makes distinguishability of X and Y-sperm difficult, and significant variation between individuals has prevented the optimization of the method [117,119,167]. Therefore, the development of an alternative sexing method that reduces sperm injury and is more affordable and practical for the equine industry and individual consumers would be beneficial.

Interestingly, some studies with human sperm have revealed that the zeta potential quality selection method has a selection bias for X-chromosome sperm [157], although others have observed no sex-bias in electrophoretic selection [168]. This implies that using this technique in horses might lead to a bias towards XX embryos, which could be beneficial in industries such as polo in which females are more desirable. Specifically, in a comparison between DGC and a combined DGC/zeta potential selection method prior to human ICSI, the DGC/zeta potential selected group resulted in 63.6% of XX babies delivered, whereas the DGC-only group only resulted in 38.5% of XX babies [157].

An altered sex ratio during electrophoretic sorting aligns with observations of a membrane charge differential between X- and Y-chromosome bearing spermatozoa; with X-chromosome bearing human sperm exhibiting approximately a −20 mV charge and Y-chromosome bearing human sperm exhibiting a charge of −16 mV [152,157]. Thus, several methods utilizing zeta potential as a basis for sex-sorting semen have been developed and are reviewed in this section.

Zeta potential sex-selection techniques include electrophoretic separation, in which fluid flow mediates sperm to swim perpendicular to an electric field. This method has been able to separate spermatozoa with a population containing almost 80% X-chromosome bearing spermatozoa [169]. Using free-flow electrophoresis, X and Y spermatozoa from humans, mice, and bulls have been successfully separated based on this external charge differential [170,171,172]. However, due to the increased net negative charge in X chromosome-bearing sperm, there have been observed biases towards the selection of X chromosome-bearing sperm due to their increased electrophoretic mobility [169,170]. A later study called the method of sex-chromosome identification via electrophoresis into question, and reported no differences in sex ratio when using electrophoretic separation [168]. No data regarding differences in zeta potential between X and Y chromosome-bearing sperm in horses has been reported.

Another recent study in donkeys observed that X chromosome bearing sperm could be successfully isolated with 90 ± 5% accuracy based on the membrane zeta potential differential between sexes and a subsequent specificity when conjugated directly to MNP under precise conditions [147]. This study utilized a modified MACS technique in which MNP were adhered to the surfaces of Y-spermatozoa using specific environmental conditions and negatively selected for X-spermatozoa [147]. Collectively, this suggests that sperm sex chromosome-dependent membrane potential, despite the poor understanding of its mechanisms, is a conserved trait across species and can be used for sex selection with equine sperm.

Regardless of the apparent conservation of sex chromosome-dependent sperm charges across species, it is unfortunate that sperm membrane charges are subject to change under a variety of physiological processes and environmental conditions. Sperm membrane composition and charge are known to change and increase, respectively, during capacitation and acrosome reaction [148,173]. It is not uncommon to see premature capacitation, or capacitation-like changes, in cryopreserved semen, and capacitation can be artificially induced using media components [174]. In addition, the interaction between sperm and specific fluids and medias may promote alternative membrane changes which can alter the surface charge [175,176,177]. Therefore, it is necessary to be consistent and critical when measuring and interpreting sperm zeta potential measures, particularly when trying to characterize minute differences between X- and Y-bearing sperm.

## 4. Conclusions

Although a variety of techniques exist to separate and select for spermatozoa based on quality and viability, as well as sex chromosome, not all methods have yet been translated to the equine industry. As the implementation of IVP in horses expands, so does the need for highly affordable and efficient semen selection techniques. The inconsistent efficacy of traditional sperm selection methods, such as SU and DGC, presents a barrier to the optimization of IVP. The introduction of techniques, such as MF, that are used clinically in other species may consequently improve IVP in the horse. There is also a need to assess the efficacy of newly developed sperm selection methods, such as viability sorting with MNP, zeta potential quality sorting, and zeta potential sex sorting, in the stallion to determine their potential for use in clinical settings. Ultimately, the rapid expansion of novel semen selection techniques provides many opportunities for improved fertilization, embryo development, and pregnancy rates within the equine breeding industry over the following decade.

## Figures and Tables

**Table 1:** Sperm selection techniques select for a variety of sperm parameters and are capable of reducing percentages of negative factors within a sample, although some methods may have detrimental effects.

Method	Selects Based On:	Benefits:	Detriments
Density Gradient Centrifugation	Morphology Cell density Motility	Enriches for: Morphology; Motility; Viability; Mitochondrial membrane integrity; Pregnancy rates; DNA integrity Removes cell and protein debris	Toxicity of Percoll^®^ Centrifugation causes DNA damage
Swim Up	Progressive motility	Enriches for: Motility; Morphology; DNA integrity Removes cell and protein debris	Low recovery rate
Density Gradient-Swim Up	Morphology Cell density Motility	Enriches for: Motility; Morphology; DNA integrity Removes pathogens	Toxicity of Percoll^®^ Centrifugation causes DNA damage
Glass Wool Filtration	In vivo fertility Motility	Enriches for: Motility; Morphology; Chromatin Condensation; Membrane, Integrity; Cleavage rates; Blastocyst rates; Pregnancy rates High Recovery Rate	Possible damage to sperm head and acrosome ultrastructure Glass wool contamination of final product
Fluorescent Activated Cell Sorting	Variable physiological markers (membrane integrity, apoptotic markers, mitochondrial membrane potential, sex chromosome)	Enriches for: Pregnancy rates; Live birth rates Removes unwanted cells	May cause oxidative and DNA damage Mechanical Stress Time consuming High operating expenses Inability to select for numerous factors
Microfluidic Sorting	Motility Rheotactic, Chemotactic, and Thermotactic behavior	Enriches for: Motility; Viability; DNA integrity Reduced ROS generaton Removes extracellular debris Can be combined with IVF	May impose stress May reduce viability in some species
Magnetic Activated Cell Sorting	Variable physiological markers (removes sperm with apoptotic markers, acrosome reacted sperm)	Enriches for: Motility; Viability; Morphology; Survival, motility, and mitochondrial integrity after cryopreservation; Sperm binding rates; Fertilization rates; Embryo development rates	May possess cytotoxic effects
Zeta Potential Selection	Greater net negative membrane charges	Enriches for: Maturity; Morphology; DNA integrity; Protamine content; Fertilization rates; Pregnancy rates	Not shown to increase motility and viability
